# Solution-free and simplified H&E staining using a hydrogel-based stamping technology

**DOI:** 10.3389/fbioe.2023.1292785

**Published:** 2023-11-09

**Authors:** Jinho Kim, Woongsun Choi, Dahyeon Yoo, Mijin Kim, Haeyon Cho, Hyun-Jung Sung, Gyuheon Choi, Jisu Uh, Jinseong Kim, Heounjeong Go, Kyung-Hak Choi

**Affiliations:** ^1^ Noul Co., Ltd., Yongin-si, Republic of Korea; ^2^ Department of Pathology, Asan Medical Center, University of Ulsan College of Medicine, Seoul, Republic of Korea

**Keywords:** agarose, cancer, eosin, hematoxylin, histopathology, hydrogel, solution-free staining, tissue staining

## Abstract

Hematoxylin and eosin (H&E) staining has been widely used as a fundamental and essential tool for diagnosing diseases and understanding biological phenomena by observing cellular arrangements and tissue morphological changes. However, conventional staining methods commonly involve solution-based, complex, multistep processes that are susceptible to user-handling errors. Moreover, inconsistent staining results owing to staining artifacts pose real challenges for accurate diagnosis. This study introduces a solution-free H&E staining method based on agarose hydrogel patches that is expected to represent a valuable tool to overcome the limitations of the solution-based approach. Using two agarose gel-based hydrogel patches containing hematoxylin and eosin dyes, H&E staining can be performed through serial stamping processes, minimizing color variation from handling errors. This method allows easy adjustments of the staining color by controlling the stamping time, effectively addressing variations in staining results caused by various artifacts, such as tissue processing and thickness. Moreover, the solution-free approach eliminates the need for water, making it applicable even in environmentally limited middle- and low-income countries, while still achieving a staining quality equivalent to that of the conventional method. In summary, this hydrogel-based H&E staining method can be used by researchers and medical professionals in resource-limited settings as a powerful tool to diagnose and understand biological phenomena.

## 1 Introduction

Tissue specimens obtained from humans can be examined using histopathological methods, which require that the samples are meticulously mounted on glass slides using a well-defined protocol, followed by microscopic analysis to identify specific pathological features. Given the inherent transparency of tissue slides, pathologists use specific staining techniques to impart contrast, facilitating visual observation and analysis (Alturkistani et al., 2015). Among the various staining methods available, hematoxylin and eosin (H&E) staining is preferred for visualizing cellular arrangements and elucidating morphological changes within tissues. Indeed, this method effectively discriminates between the nucleus and cytoplasm, thereby enabling comprehensive examination and analysis (Fischer et al., 2008; Feldman and Wolfe, 2014). Owing to its broad applicability across diverse tissue types and cost-effectiveness, H&E staining represents the foremost and foundational staining technique employed in most histopathology laboratories.

H&E staining typically involves four distinct sequential steps: hematoxylin staining to selectively stain the nuclei, differentiation that serves to decolorize excess hematoxylin, bluing to facilitate the conversion of the hematoxylin stain color to blue, and eosin staining to stain the cytoplasmic components. However, this staining procedure also requires multiple sequential washing steps between the four staining stages using a solution predominantly composed of water. Although H&E staining is a fundamental histopathological technique, its practical implementation can pose significant challenges in low- or middle-income countries due to impaired access to water, insufficient sewage facilities, and limited wastewater management systems (Adesina et al., 2013; Ntiamoah et al., 2019). Furthermore, constrained settings lacking trained technicians responsible for performing staining procedures and the potential for cross-contamination via the staining agents can significantly diminish the diagnostic precision achieved through H&E staining (Benediktsson et al., 2007; Fleming et al., 2017). Despite these challenges, the most important aspect of a diagnosis is the staining quality, which is why the process has been used without modification. Moreover, these limitations can be addressed in well-supported hospital institutions by implementing automated slide stainers for error minimization. Recently, numerous studies have emerged wherein deep learning methodologies have been employed to investigate virtual H&E staining approaches, eliminating the need for conventional physical staining techniques (Borhani et al., 2019; Rivenson et al., 2019; Pradhan et al., 2021) Virtual H&E staining offers a rapid, cost-effective, and chemical-free alternative for histopathology; however, further research is necessary to enhance the correlation and accuracy between virtually stained tissues and their actual dyed counterparts, considering potential artifacts or aberrations that may arise from the virtual staining process (Bai et al., 2023). Hence, there is an urgent need for technologies that can be effectively implemented in real-world scenarios to overcome the current technical limitations.

In this study, we describe a novel method for H&E staining that employs hydrogel patches and is entirely devoid of water aiming to simplify the standard eight-step procedure to only two steps, while ensuring the accuracy of the histopathological analysis. In summary, the herein-proposed hydrogel-based H&E staining method is expected to overcome the need for water facilities for conventional solution-based stain and the fundamental challenges faced by low- and middle-income countries, while it also can be coupled with available technology for digital pathology analysis.

## 2 Materials and methods

### 2.1 Fabrication of hydrogel patches

A commercialized regressive hematoxylin solution (CV Select HemaMAX; BBC Biochemical, Mount Vernon, WA, United States) was used to fabricate hematoxylin patches, which included hematein, mordant, and preservative components to provide a stable state and pH regulation. A 2.5% (w/v) low-electroendosmosis agarose powder (Genomics One Corp., Ottawa, Canada) was mixed with Milli-Q water up to 100 mL of the final solution and heated at 100°C. Hematoxylin solution (20% v/v) and Tween-20 (2% v/v) (Sigma-Aldrich, St. Louis, MO, USA) were added to the mixture and stirred at 200 rpm for 2 min. The resulting mixture was poured into dedicated patch holders and allowed to solidify at room temperature at 25°C (Supplementary Figure S1). The fabricated hematoxylin patch had a pH of 2.75, which is appropriate for the isoelectric point of hematein.

To fabricate the eosin patches, 0.075% (w/v) eosin Y disodium salt powder (Sigma-Aldrich) was mixed with 100 mL Milli-Q water and 50 mM Bis-Tris buffer (Sigma-Aldrich). The pH was adjusted to 6.0 using 99.5% acetic acid (Sigma-Aldrich) and 1M sodium hydroxide (Sigma-Aldrich). The prepared mixture was heated at 100°C and ethylene glycol (2% v/v) (Sigma-Aldrich) detergent was added while stirring. The final mixture was poured into a dedicated holder and solidified at room temperature at 25°C (Supplementary Figure S1).

### 2.2 Formalin-fixed paraffin-embedded (FFPE) tissue samples

Multiple unstained 4-μm thick sections were made from 10% FFPE tissue blocks of resection specimen of colon adenocarcinoma, papillary thyroid carcinoma, hepatocellular carcinoma, and malignant lymphoma (lymph nodes) from cancer patients. This study was approved by the Institutional Review Board of the Asan Medical Center (No. 2019-1174).

### 2.3 Conventional solution staining

FFPE tissues were stained on a Sakura Tissue-Tek Prisma Automated Slide Stainer (Sakura Finetek, Alphen Aan Den Rijn, Netherlands). Sections were deparaffinized with xylene (Duksan, Gyeonggi-do, South Korea) and rehydrated thrice with 100% ethanol (Duksan). The rehydrated sections were then washed under tap water for 3 min and stained with progressive Tissue-Tek hematoxylin solution (Sakura Finetek) for 115 s. The sections were rinsed with tap water for 3 min and stained with Tissue-Tek eosin solution (Sakura Finetek) for 15 s. The sections were then rinsed with distilled water for 10 s and dehydrated five times in 100% ethanol. The sections were cleaned with xylene and cover-slipped (Tissue-Tek Glass coverslipper, Sakura Finetek).

### 2.4 Hydrogel patch staining

In the tissue processing step, similar to conventional solution-based H&E staining, FFPE tissue slides were deparaffinized using xylene (Duksan) and rinsed with 100% ethanol (Duksan) to remove the residual xylene solution. However, for the hydrogel patch, there was no need for sequential hydration using ethanol concentrations below 95%. After removing xylene with 100% ethanol, the slides were dried at room temperature to rapidly evaporate the residual ethanol solutions from the tissue. Following tissue processing, H&E staining was performed using a series of simple stamping steps with the two hydrogel patches containing the H&E dyes. For post-processing, after staining, the residual staining solution on the tissue was removed with 100% ethanol, without needing a gradual increase in ethanol concentration for dehydration as in the conventional method. A clearing process was performed using xylene. Once all the steps were completed, the slides were mounted with 70 μL of mounting medium (Permount SP15-100, Fisher Scientific, Pittsburgh, PA, USA) and coverslipped with a 24 × 50 mm cover glass (Paul Marienfeld, Lauda-Königshofen, Germany) of 0.13–0.16 mm thickness. The entire H&E staining protocols using conventional solution staining and hydrogel patch methods are compared in Supplementary Table S1.

### 2.5 Microscopy imaging

An upright microscope (CX33; Olympus Live Science, Tokyo, Japan) with a 20× lens (UPLXAPO20X; Olympus Live Science) was used to observe the stained tissues. Digital images with a resolution of 5,440 × 3,648 pixels were acquired using Excope software (Olympus Live Science). To ensure consistent image acquisition conditions, the illuminator and condenser settings were standardized to have identical physical parameters. Additionally, the image capture software was configured to maintain consistent digital values for brightness, hue, and other parameters.

### 2.6 Stained color quantification

Colorectal cancer tissues, which allowed relatively easy confirmation of morphological structures, were used to quantitatively analyze the colors of the H&E-stained tissues. Owing to the characteristics of the tissue slide preparation method for FFPE tissue samples, it was not possible to create identical tissue sections with the same structure; nonetheless, morphologically similar tissue sections were used to enable accurate quantitative comparisons. Specifically, we conducted quantitative analysis of smooth muscle tissues within colorectal cancer, which exhibited low heterogeneity and a uniform structure. The acquired digital images were analyzed using the free open-source ImageJ software (https://imagej.nih.gov/ij/index.html). Briefly, instead of separating the hematoxylin and eosin signals, we converted the red, green, blue colors to grayscale and extracted their values for analysis (Supplementary Figure S3) (Azevedo Tosta et al., 2019; Chlipala et al., 2020): when the staining became lighter, the overall color became brighter, resulting in grayscale values close to 255 (white), whereas the values decreased and approached zero (black) as the staining was darker. However, the diversity of the background surface area on the slide, which lacks the tissue layer being stained, can introduce errors in the grayscale values owing to variability in tissue morphology. Therefore, we also reduced analysis errors by extracting the hue, saturation, and brightness values of the background from the images (which were acquired with identical physical and digital settings) while focusing exclusively on the tissue area of interest (Supplementary Figure S3). The mean densitometric values of the entire tissue were calculated as follows:

$$Mean\;grayscale\;value\;of\;interest=\frac{Sum\;of\;grayscale\;value}{Sum\;of\;pixels}\times \left(Total\;area-Background\;area\right)$$

### 2.7 Semi-quantitative analysis

The stained slides were scanned at 40× magnification using a whole-slide scanner (Pathology Scanner Second Generation SG300; Philips, Amsterdam, Netherlands). Five pathologists reviewed the slides using the Image Management System Viewer software (Philips). For comparative analysis, 10 unstained sections of colon adenocarcinoma were randomly selected to be stained using the hydrogel patch method or the solution-based auto stainer Sakura Tissue-Tek Prisma system (Sakura Finetek). Five trained pathologists from the Asan Medical Center, a tertiary general hospital reviewed the slides on a 1–5 scale according to five evaluation criteria (see Supplementary Material). Each criterion was designed to evaluate the quality of hematoxylin and eosin staining, overall staining quality, and defects presence and the scores were assigned based on the evaluation level for each criterion (College of American Pathologists [CAP] and National Society for Histotechnology [NSH], 2023; Kumar and Kiernan, 2010). The Mann–Whitney U test was used for statistical analysis and *p*-value <0.05 was deemed significant.

## 3 Results

### 3.1 Hydrogel patch staining

**FIGURE 1:**
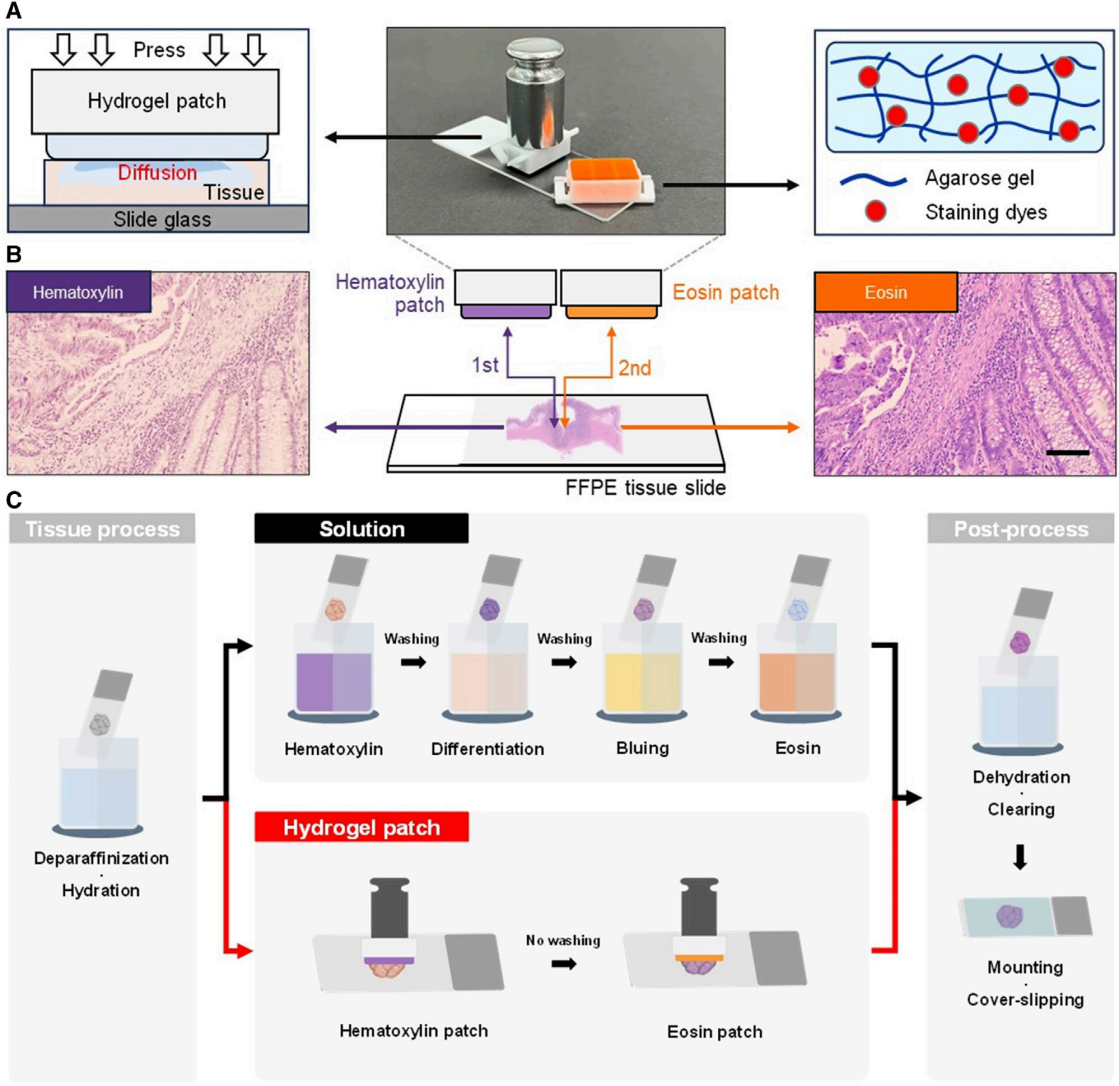
Photographs and schematic diagrams of a hydrogel patch and working principle. **(A)** Hydrogel patch stamping process that allows the diffusion of stain into the tissue (left). The hydrogel patch is composed of an agarose network and the solution contains the staining dyes in the agarose pores (right). **(B)** Schematic diagrams of the staining process using hematoxylin and eosin patches. The agarose hydrogel patches are applied to the tissue section in a sequential stamping process to induce staining. Photographs showing the color change of colorectal tissue samples after the stamping of each patch. Scale bar: 100 µm. **(C)** Schematic comparative diagram of overall H&E staining process using the solution-based and hydrogel patch-based stamping staining method on formalin-fixed paraffin-embedded (FFPE) tissue sections.

The hydrogel patch was easily fabricated by preparing an agarose solution mixed with the respective staining dye (hematoxylin or eosin), which was then solidified in plastic holders with 11 × 18 mm (staining area) (Supplementary Figure S1). When the surface of the hydrogel patch containing the staining dye within the agarose microstructure was put in contact with the tissue, diffusion occurred due to the increased surface:volume ratio between the staining solution and the tissue, resulting in tissue staining (Figure 1A) (Bae et al., 2023). Additionally, an external force of a weight of 50 g was applied to the upper part of the patch to accelerate the discharge of the staining solution and enhance diffusion. Hence, H&E staining of FFPE tissue slides using the hydrogel patch method was achieved with two steps, as compared with the standard multistep H&E solution approach (Figures 1B,C).

The advantages of the hydrogel patch included solution-free staining, omission of washing steps, and skipping the differentiation and bluing processes, thereby simplifying the overall H&E staining process. Furthermore, in tissue processing and post-processing of FFPE tissue sections, the hydrogel patch allowed the omission of the hydration and dehydration processes based on a progressive concentration of ethanol below 95%. Thus, this approach eliminated the need of water, making it applicable to environments with limited resources. By simplifying the overall conventional method from 20 (Schmitz et al., 2010; Cardiff et al., 2014; Feldman and Wolfe, 2014) to 10 steps (Figure 1C and Supplementary Table S1), the staining process became less challenging.

### 3.2 Characterization of the hydrogel staining conditions

**FIGURE 2:**
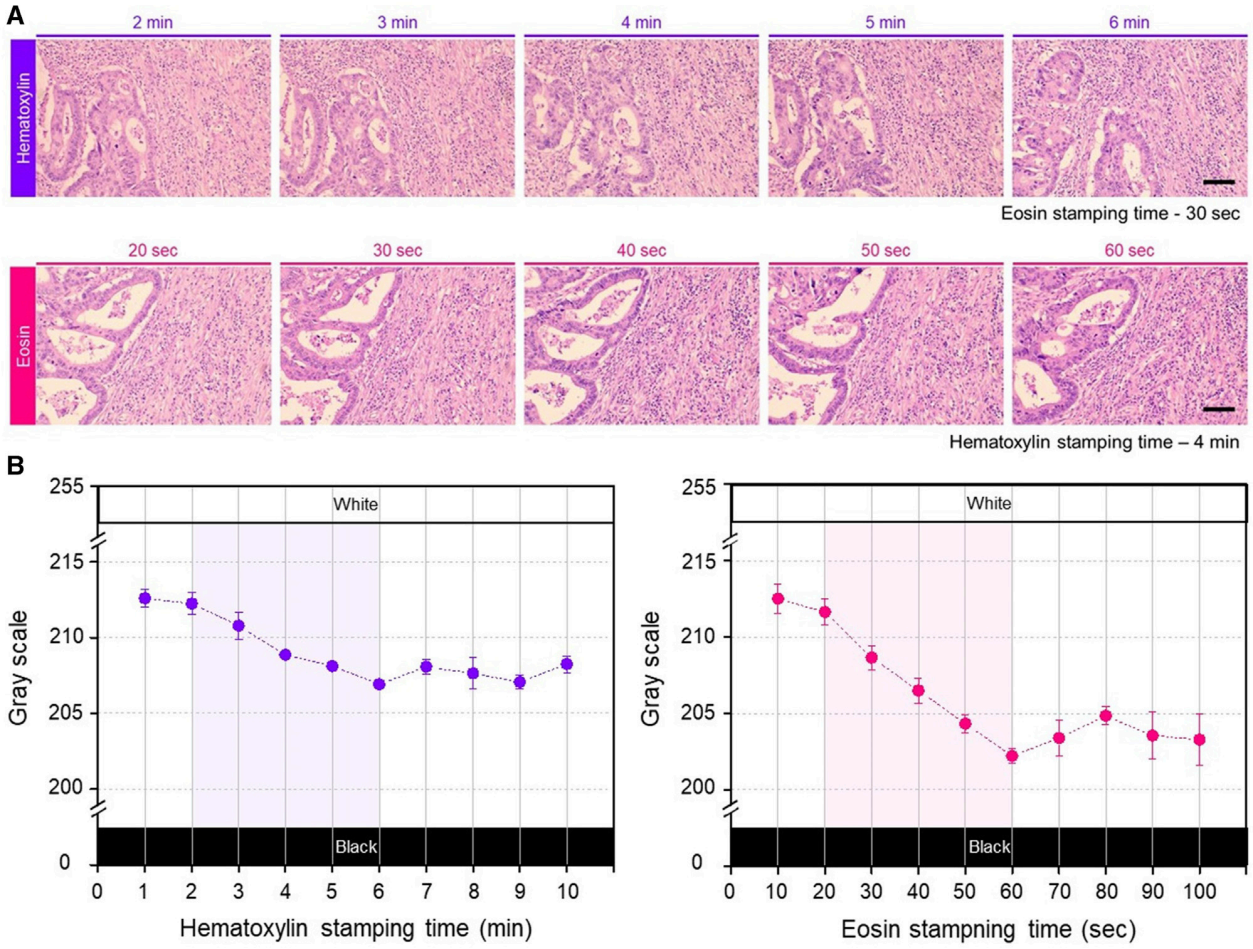
**(A)** Photographs of the gradual change of H&E stained color according to change in the patch stamping time. Each row shows the gradual color changes obtained with increasing staining time of each hydrogel patch. The stamping time for the counterstain hydrogel patch was consistently maintained fixed. Scale bar: 100 µm. **(B)** Quantitative analysis results by gray scale variation according to the stamping time of each patch, repeated three times for each staining condition. The shaded areas in each graph represent linear and applicable range.

To characterize the staining conditions of the hydrogel patches, the staining performance was analyzed based on the staining time of colorectal cancer tissues under identical conditions. To evaluate staining performance, the stamping time for the counterstained patches was maintained consistent across the entire measurement range. Cells stained with purple hematoxylin could be used to identify the mucosa, submucosa, and cancer tissue, whereas the remaining parts stained with pinkish eosin, including muscle and normal tissue, could be distinguished (Supplementary Figure S2). Notably, the overall color of the tissue became qualitatively darker with increasing staining time (Figure 2A). Next, for a more detailed analysis, quantitative analysis of the color changes was performed based on grayscale values, excluding blank areas without tissue while focusing on the smooth muscle, which exhibited the least heterogeneity in the colorectal structure (Supplementary Figure S3). For the hematoxylin hydrogel patch, changes in the staining color were observed within a staining time of 1–10 min (with a fixed eosin staining time of 30 s), showing a linear color-change pattern between 2 and 6 min; however, staining saturation was observed after 6 min (Figure 2B, left). Similarly, staining with the eosin patch showed a linear color-change pattern between 20 and 60 s, with color saturation being observed after 60 s within a staining time range of 10–100 s (with a fixed hematoxylin staining time of 4 min) (Figure 2B, right). Since eosin staining affected most areas of the tissue (except the nucleus), lower grayscale values were detected with increasing staining time compared with those obtained with hematoxylin staining (Figure 2B). Additionally, in H&E staining, eosin acts as a counterstain to stain cells based on hematoxylin staining for diagnostic purposes; therefore, to control hematoxylin staining for a longer duration and allow for a more precise adjustment of hematoxylin staining, the concentration of the patch was adjusted.

### 3.3 Evaluation of hydrogel staining outcome

To assess the clinical applicability of the optimized hydrogel patch, a semi-quantitative evaluation was conducted by trained pathologists using five preestablished criteria (see Supplementary Material), using a scoring scale ranging from 1 to 5 points (Supplementary Table S2). Comparison of the outcome of each staining method showed that the staining quality of hematoxylin (Q1) and eosin (Q2), and overall staining contrast (Q3) were superior when using the conventional solution-based automated slide stainer compared with the hydrogel patch method (Table 1). Nevertheless, no significant difference in the staining uniformity (Q4) was observed between the two methods (Table 1). This finding indicates that the hematoxylin and eosin dyes diffused uniformly across the entire tissue section corresponding to the hydrogel patch area, resulting in stable and consistent staining. Moreover, similar results were obtained with both methods regarding non-specific staining patterns on the background (Q5) (Table 1); considering that the hydrogel patch method did not require rising steps, it achieved a better outcome than the solution method. Taken together, these results suggest that, despite omitting the washing step, the hydrogel patch method effectively releases an appropriate amount of solution. Simultaneously, it absorbs any residual solution, enabling clear staining of the entire tissue section without leaving any remnants.

**TABLE 1:** Comparative evaluation of the staining outcome of the hydrogel patch and conventional solution-based methods as scored by trained pathologist.

Evaluation criteria	Solution^a^	Hydrogel patch^a^	*p*-value^b^
Q1. Hematoxylin staining	4.20 ± 0.06	3.76 ± 0.07	0.014
Q2. Eosin staining	4.40 ± 0.06	3.40 ± 0.09	0.011
Q3. Hematoxylin and eosin contrast	4.68 ± 0.08	3.24 ± 0.10	0.010
Q4. Staining uniformity	4.24 ± 0.07	4.16 ± 0.07	0.507
Q5. Background staining	4.48 ± 0.05	4.68 ± 0.08	0.097

^a^ Data are presented as mean ± standard error of the mean.

^b^ Statistical analysis was performed using the Mann–Whitney *U* test.

### 3.4 Hydrogel-based method outcome concerning staining artifacts

In histopathological examinations using H&E staining, several factors, collectively known as “artifacts,” can lead to inadequate and inconsistent tissue quality. These factors include fixation, tissue processing, staining, and contamination, and they can interfere with accurate diagnosis by pathologists (Rastogi et al., 2013; Taqi et al., 2018). Among these artifacts, variations H&E staining colors are primarily influenced by the thickness of the sample and they also occur due to differences in tissue preparation between medical centers (Falahkheirkhah et al., 2022). To overcome these staining artifacts, post-processing adjustments can be implemented to standardize the obtained images (Yagi, 2011; Azevedo Tosta et al., 2019). Nonetheless, to physically reduce the inherent staining color differences can be more effective than these post-processing adjustments. To achieve this, the adjustment of various factors, such as hydration, staining, differentiation, and bluing, is required, making H&E staining a cumbersome and challenging process. Furthermore, in settings that utilize automated slide stainers capable of high-capacity staining, such as tertiary general hospitals, it is difficult to change the protocols for each staining procedure.

**FIGURE 3:**
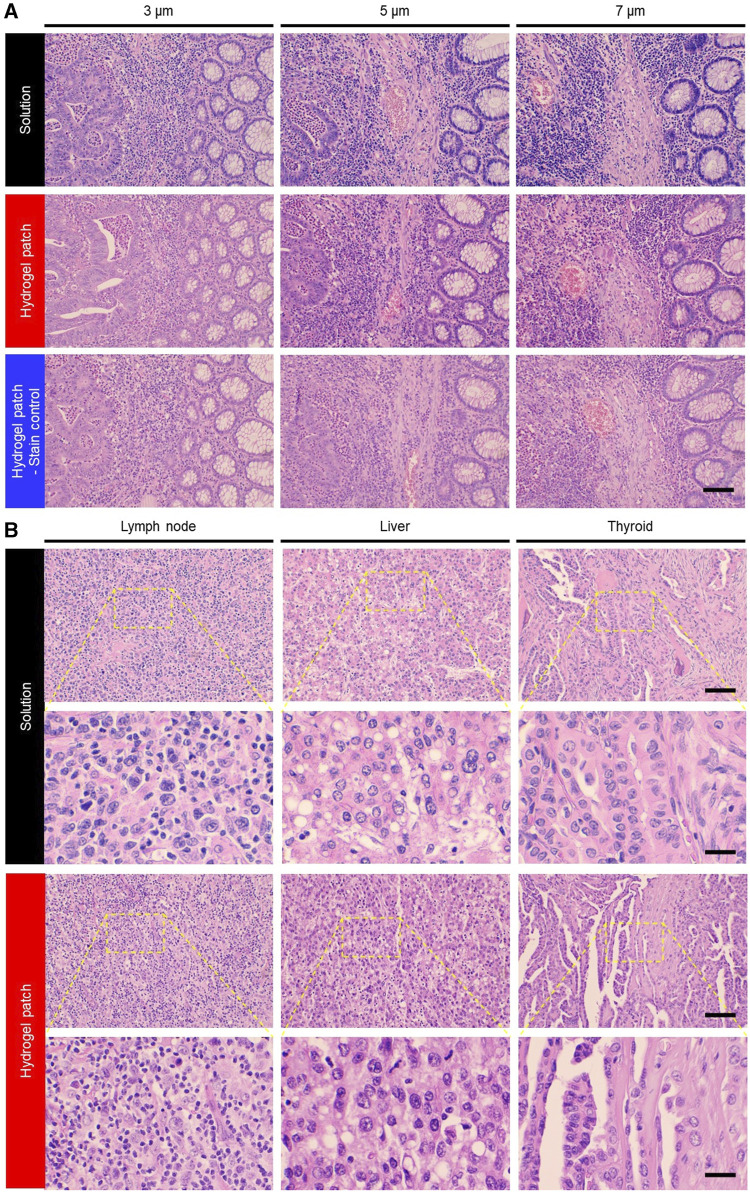
**(A)** Photographs of variations in staining color due to sample thickness artifacts associated with different staining methods. This staining limitation was overcome by adjusting the hydrogel patch stamping time (3rd row). Scale bar: 100 µm. **(B)** Photographs of colon adenocarcinoma, malignant lymphoma, papillary thyroid carcinoma, and hepatocellular carcinoma tissues from cancer patients stained with H&E using solution and hydrogel patch. Scale bars: 100 (top row) and 25 (bottom row) µm.

Since staining artifacts can effectively impair the diagnostic potential of H&E staining, we evaluated the outcome of the hydrogel patch regarding changes in tissue thickness. Overall, an increase in tissue thickness resulted in the absorption of a greater amount of staining dye, leading to darker staining colors of the target tissue components (2nd row), but these color changes were similar to those seen with the solution-based staining method (1st row) (Figure 3A). Further analysis of sectional images of 7-μm thick stained samples confirmed that the color variations observed with both staining methods were primarily explained by thickness artifacts rather than by inherent staining method characteristics, as no color differences were detected among the upper and lower tissue layers (Supplementary Figure S4). Based on these results, we could easily compensate for the color differences in the staining caused by thickness artifact (Figure 3A, 3rd row) by varying the stamping time of the two patches.

Next, the diversity of the staining colors in FFPE tissue slides prepared using unique tissue preparation protocols from three different medical institutions were evaluated. The tissue samples showed a pattern similar to that of the conventional solution-based method, indicating the presence of institutional artifacts (Supplementary Figure S5, 1st and 2nd rows). However, compared with the conventional solution-based staining method, which requires adjusting and combining numerous staining step parameters, overcoming these artifacts with the hydrogel patch method was made easier by adjusting only one parameter, namely, stamping time (Supplementary Figure S5, 3rd row).

Finally, based on previously established hydrogel-based staining conditions using colorectal cancer tissues, other tissue types (namely, lymph nodes, liver, thyroid, and pancreas samples) were stained using hydrogel patches. The staining results showed the feasibility of staining various tissue types using this new simplified method (Figure 3B). Moreover, it was indicated that hydrogel patches could be used for staining not only FFPE-processed tissues but also frozen-processed tissues (Supplementary Figure S6). This demonstrates the versatility of the hydrogel patch staining method across different tissue states and the potential to overcome tissue preparation or artifacts.

## 4 Discussion

The H&E staining protocol is widely used in pathology and histological laboratories; however, is a complex and time-consuming diagnostic method. To overcome these limitations, we developed a hydrogel-based solution-free H&E staining approach. By using only two hydrogel patches, the staining process was dramatically reduced, saving time and simplifying the process, while retaining the ability to discriminate the different tissue features.

Achieving a proper balance of colors between the hematoxylin and eosin stains enhances the visibility of tissue morphology. The hematoxylin patch contained a mordant and mature hematein at a pH of 2.6, which enabled the staining of negatively charged nuclei within the tissue (Kumar and Kiernan, 2010; CAP and NSH, 2023). Generally, regressive hematoxylin staining requires a differentiation step due to the rapid overstaining of the samples within a short period of time (Llewellyn, 2009). Noteworthily, as the staining concentration in the patches, as well as the diffusion of the dyes into the tissues, could be easily adjusted, this new hydrogel-based staining approach prevented the saturation of the samples (Bae et al., 2023), regardless of the staining time and elimination of the differentiation step. The eosin patch contained the positively charged eosin Y dye at a pH of 6.0, which binds to proteins, the cytoplasm, and other components (such as red blood cells) in tissues, excluding the nuclei. Eosin is known to exhibit optimal staining characteristics at pH 4.6–5.0 (Kumar and Kiernan, 2010; CAP and NSH, 2023). By applying the eosin patch at pH 6.0 to the tissue surface, which has a pH of 2.75 after hematoxylin patch staining, the tissue maintains a mean pH of 4.7–4.9, providing an appropriate staining pH environment for eosin staining and thus enhancing staining efficiency. Additionally, owing to the pH of the patch being 6.0, the bluing effect of hematoxylin, caused by the conversion of hematoxylin into an insoluble form, shifting its color from red to blue, (Llewellyn, 2009), could be applied simultaneously with the eosin staining.

Hydrogels based on agarose are easy to manufacture and manipulate, making them readily accessible. Of note, this hydrogel-based staining method allows solution-free staining and adjustment by stamping time, even in the presence of tissue artifacts and variations in tissue preparation. These advantages were further confirmed by trained pathologists, as demonstrated by similar staining uniformity and background staining outcomes as compared with the standard H&E solution method. The staining qualities of H&E, along with the resulting contrast differences, tend to vary according to the preferences of the pathology institutions, observers, or staining equipment used. Notably, based on the characterization results of the hydrogel staining conditions, the staining color can be easily adjusted through the stamping time, allowing for customization according to user preferences. Herein, we demonstrated the application of a hydrogel-based H&E staining method to frozen tissue slides. Diagnosis using frozen sections is a necessary pathological examination performed during surgery to achieve a turnaround diagnosis time within 20 min. However, frozen tissue sections have a thickness of 5 μm and are commonly inconsistently sectioned owing the nature of frozen samples, leading to artifacts that can lower the quality of slides and cause variations in H&E staining, making diagnosis further challenging (Rastogi et al., 2013; Dey, 2018; Taqi et al., 2018). Therefore, the application of a hydrogel staining method that enables uniform staining and easy adjustment is expected to significantly enhance the diagnostic accuracy, particularly in frozen tissue samples.

Several attempts have been made to use digital pathology in H&E staining with the aid of artificial intelligence (Janowczyk and Madabhushi, 2016; Clarke and Treanor, 2017; Niazi et al., 2019). However, the diversity of H&E staining poses a challenge to the application of machine learning algorithms (Madabhushi and Lee, 2016; Komura and Ishikawa, 2018). In particular, the presence of residual staining in the background can lead to increased imaging time and file size in whole-slide scanning because the scanner recognizes the background as a stained area (Magee et al., 2009). Artificial intelligence techniques also pose challenges to image recognition using artificial intelligence techniques. To address this issue, complementary methods, such as generative adversarial networks (Zhu et al., 2017; Shaban et al., 2019), DeepFocus approaches (Senaras et al., 2018), and score-based diffusion models (Jeong et al., 2023), have been applied to digitally adjust the color of whole-slide images for standardization. However, there are still limitations and there is a growing need for new staining methods with minimal color variations in tissues. This hydrogel-based H&E staining approach provides a new foundation to enhance H&E-based diagnosis; nonetheless, additional investigations can still be made. For example, extending the application of hydrogel-based H&E staining may rely on simplifying the pre- and post-processing steps of deparaffinization and clearing FFPE tissue slides in the format of patches, thereby creating a complete solution-free method. In addition, fully automating the serial stamping processes would prevent even the slightest possibility of handling errors, allowing non-experts to achieve high-quality uniform staining.

## 5 Conclusion

We developed a hydrogel patch-based method for H&E staining that can achieve a staining quality comparable with that of the gold-standard solution method while being simple to manufacture, solution-free, and simplified in its steps. Moreover, it can be applied even in resource-limited middle- and low-income countries, offering a powerful tool that provides easier access for researchers and medical professionals to explore pathological and biological phenomena.

## Data Availability

The original contributions presented in the study are included in the article/Supplementary Material, further inquiries can be directed to the corresponding authors.
