# Supplementary material for: Solution-free and simplified H&E staining using a hydrogel-based stamping technology
Source: Front Bioeng Biotechnol. 2023 Nov 9;11:1292785. doi: 10.3389/fbioe.2023.1292785 (PMC10665566; doi:10.3389/fbioe.2023.1292785)
Supplement: Supplementary file 1 [file DataSheet1.PDF]

Supplementary Material

1 Supplementary Methods

1.1 Semi-quantitative analysis

- Evaluation criteria:

Q1. Well-defined chromatin pattern and nuclei not smudgy or bubbling.

= Evaluation of hematoxylin staining.

Q2. Cytoplasmic and extracellular components are distinguishable and not hazy or bleeding.

= Evaluation of eosin staining.

Q3. Contrast between the nuclear stain (hematoxylin) and the cytoplasmic stain (eosin).

= Determine overall stain quality.

Q4. Uniformity of hematoxylin and eosin stain over the entire area of the section.

= Certain solutions are not sufficient or uneven in concentration to cover the entire section.

Q5. The non-specific staining patterns on background.

= The rinse steps are not adequate after staining steps to remove excess staining solution.

**Level**	**Low**	**Moderate low**	**Moderate**	**Good**	**Excellent**
Points	1	2	3	4	5

2 Supplementary Figures

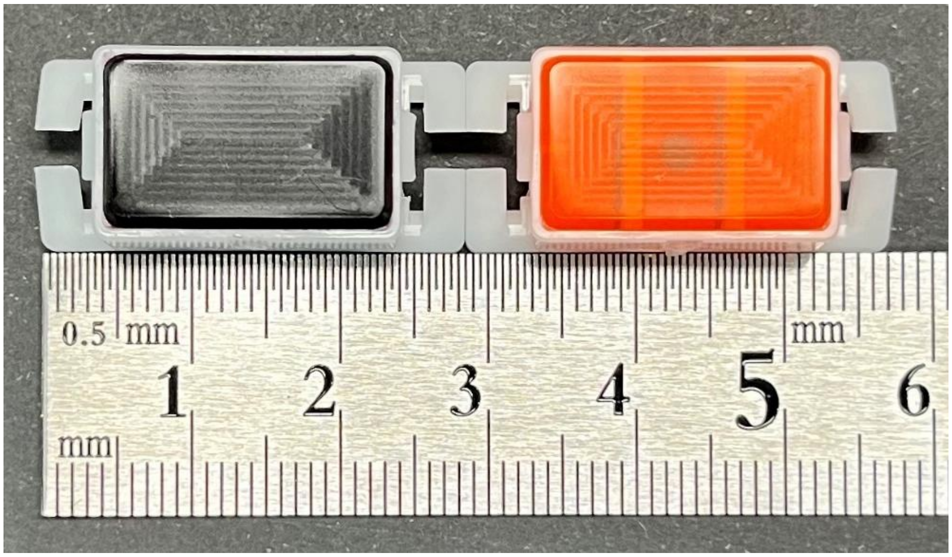

Figure S1. Photograph of fabricated hematoxylin (left) and eosin (right) patch.

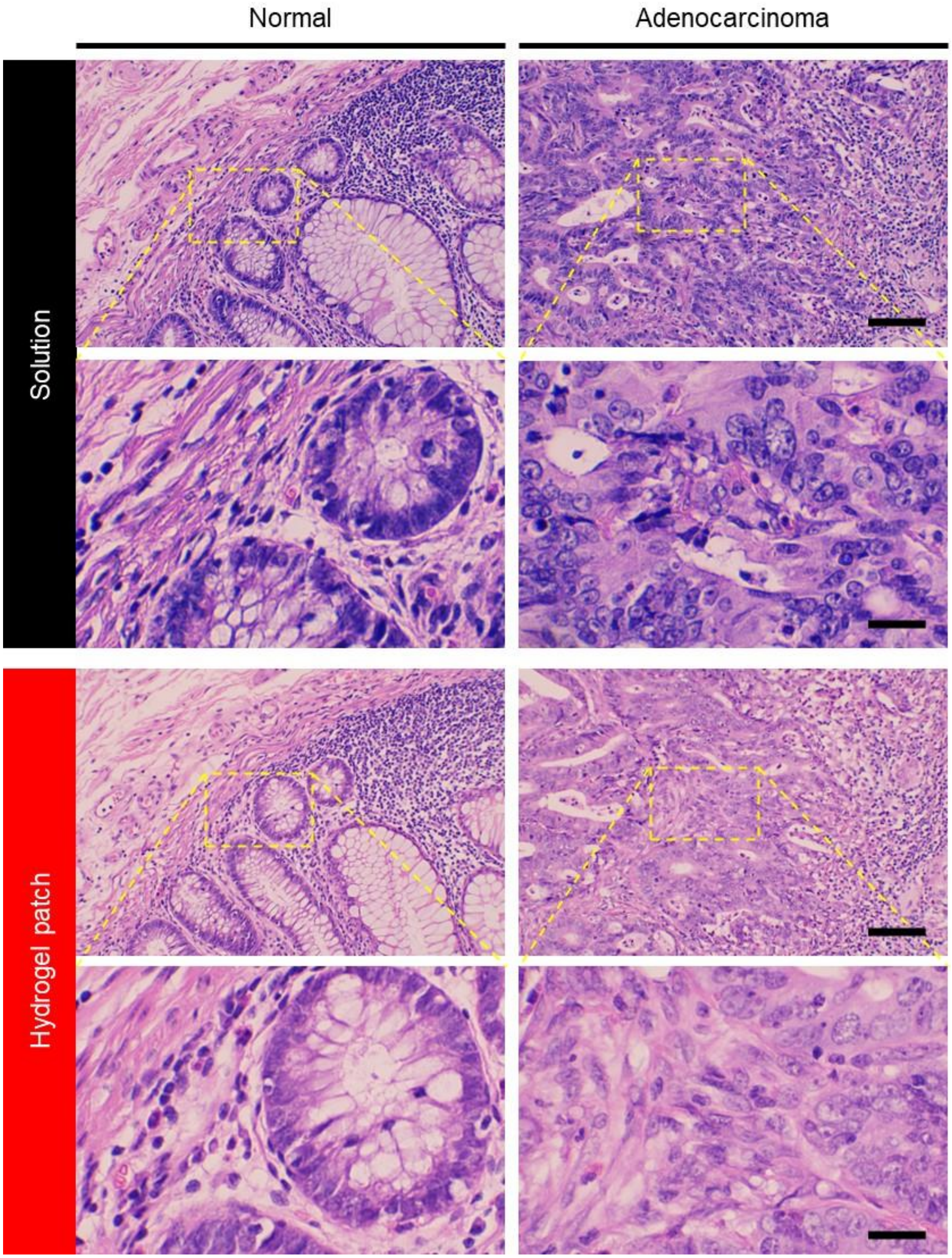

Figure S2. Photographs of comparative images between normal and adenocarcinoma sections of colorectal cancer tissues based on staining methods. Scale bars: 100 (top row) and 25 (bottom row) μm.

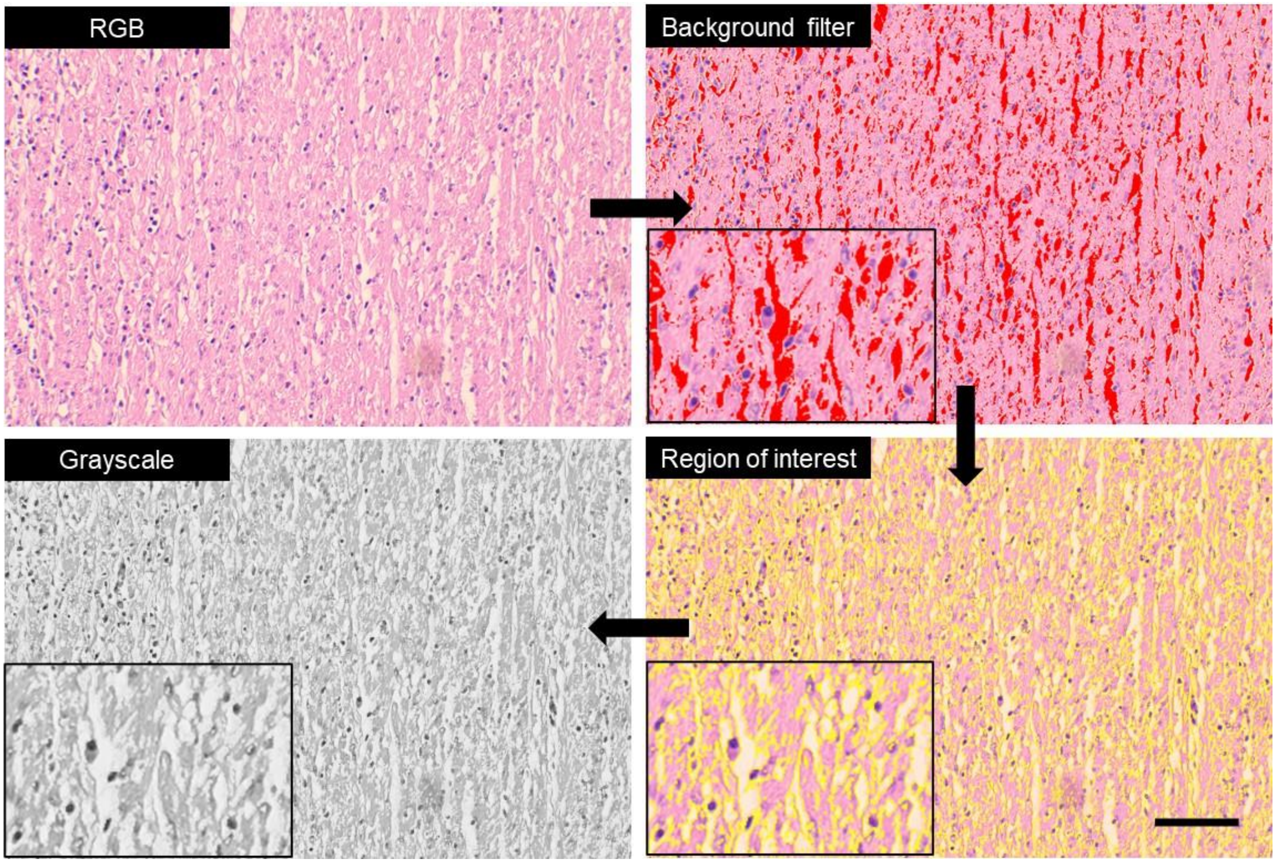

Figure S3. Image processing flowchart applied for the quantitative analysis of smooth muscle sections of colorectal cancer tissue. The background surface area (red color) was first excluded from the original red, green, and blue image, the analysis area was selected for target pixels (yellow color guideline), and then converted to grayscale. Scale bar: 100 µm.

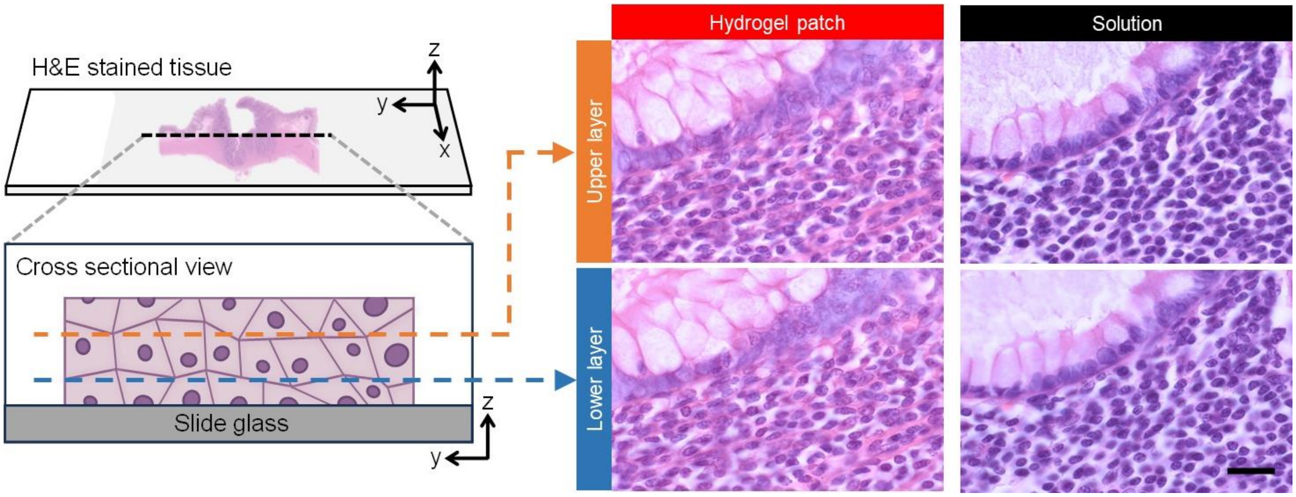

Figure S4. Photographs of colorectal cancer tissue sections (7-μm thickness) stained with H&E at different z-axis heights. Scale bar: 20 μm.

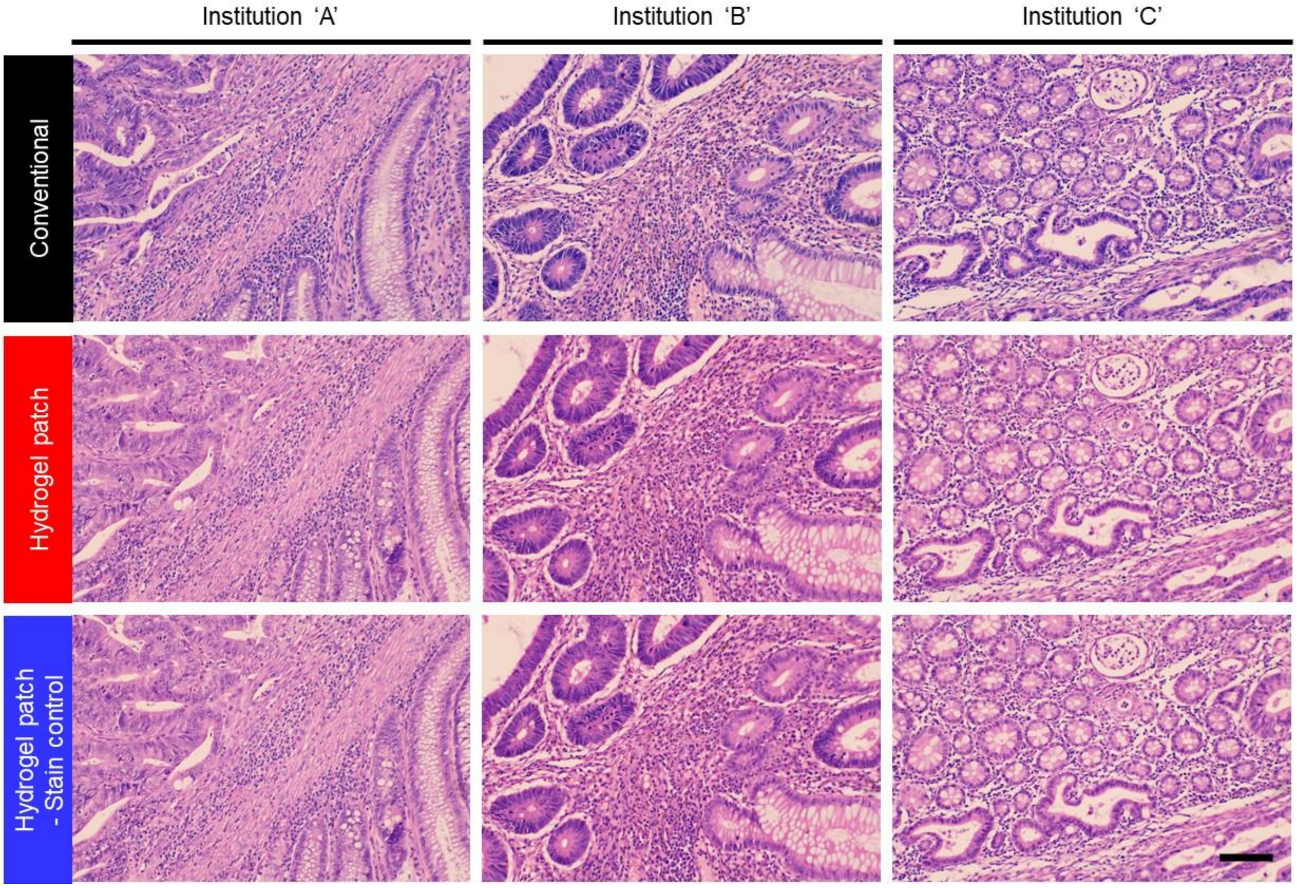

Figure S5. Photographs of variations in staining color due to artifacts caused by different FFPE tissue preparation protocol applied by different hospital institutions. This staining limitation was overcome by adjusting the hydrogel patch stamping time (3rd row). Scale bar: 100 µm

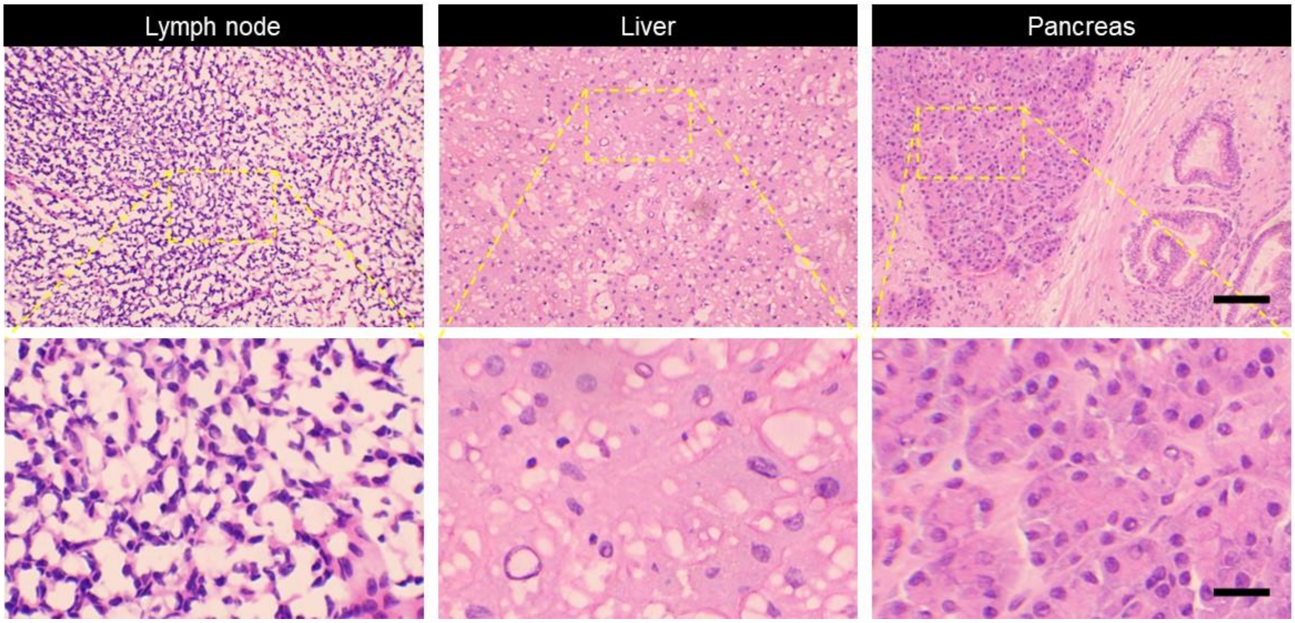

Figure S6. Photographs of frozen tissue samples from various organs stained with H&E using the hydrogel patch. Scale bars: 100 (top row) and 25 (bottom row) µm.

3 Supplementary Tables

Table S1. Comparison of H&E staining protocols for commercialized solution-based automated slide stainer and hydrogel patch staining.

Main Process	Solution based stain		Hydrogel patch stain	
	**Detail process**	**Incubation time (sec)**	**Detail process**	**Incubation time (sec)**
Deparaffinization	Xylene	240	Xylene	240
	Xylene	300	Xylene	300
	Xylene	300	Xylene	300
Hydration	Ethanol 100%	60	Ethanol 100%	60
	Ethanol 100%	60	Ethanol 100%	60
	Ethanol 95%	60	-	-
	Washing	300	Dry ethanol	300
Hematoxylin stain	Progressive hematoxylin	115	Regressive hematoxylin patch	360
Differentiation	Washing	300	-	-
Bluing			-	-
Eosin stain	Eosin	12	Eosin patch	60
	Washing	10	-	-
Dehydration	Ethanol 100%	10	Ethanol 100%	60
	Ethanol 100%	10	-	-
	Ethanol 100%	60	-	-
	Ethanol 100%	60	-	-
	Ethanol 100%	60	-	-
Clearing	Xylene	60	Xylene	60
	Xylene	60	-	-
	Total time	34 min 37 sec	Total time	30 min

Table S2. The raw data of tissue slides stained with solution and hydrogel patches evaluated by five pathologists.

	Solution	S#1	S#2	S#3	S#4	S#5	Hydrogel	S#1	S#2	S#3	S#4	S#5
Pathologist A	Q1	4	4	4	4	4	Q1	3	4	3	4	4
	Q2	4	4	4	4	4	Q2	3	3	3	3	3
	Q3	5	5	5	5	5	Q3	2	2	2	2	2
	Q4	3	2	3	2	2	Q4	3	2	3	3	2
	Q5	4	4	4	4	4	Q5	4	4	4	4	4

	Solution	S#1	S#2	S#3	S#4	S#5	Hydrogel	S#1	S#2	S#3	S#4	S#5
Pathologist B	Q1	5	5	5	5	5	Q1	4	4	4	4	4
	Q2	4	4	4	4	4	Q2	2	3	3	2	3
	Q3	5	4	4	4	5	Q3	3	3	3	3	3
	Q4	4	4	4	4	4	Q4	4	4	4	4	4
	Q5	5	5	5	5	5	Q5	5	5	5	5	5

	Solution	S#1	S#2	S#3	S#4	S#5	Hydrogel	S#1	S#2	S#3	S#4	S#5
Pathologist C	Q1	5	5	5	5	5	Q1	4	4	4	5	4
	Q2	5	5	5	5	5	Q2	4	5	4	4	5
	Q3	5	5	5	5	5	Q3	4	4	4	5	4
	Q4	5	5	5	5	5	Q4	5	5	5	5	5
	Q5	4	4	4	4	4	Q5	5	5	5	5	5

	Solution	S#1	S#2	S#3	S#4	S#5	Hydrogel	S#1	S#2	S#3	S#4	S#5
Pathologist D	Q1	3	3	3	3	4	Q1	4	4	4	4	4
	Q2	5	5	5	5	5	Q2	4	4	4	4	4
	Q3	5	5	5	5	5	Q3	3	4	4	4	4
	Q4	5	5	5	5	5	Q4	5	5	5	5	5

	**Q5**	5	5	5	5	5	**Q5**	5	5	5	5	5

	**Solution**	**S#1**	**S#2**	**S#3**	**S#4**	**S#5**	**Hydrogel**	**S#1**	**S#2**	**S#3**	**S#4**	**S#5**
**Pathologist E**	**Q1**	3	4	5	4	3	**Q1**	3	3	3	3	3
	**Q2**	3	4	5	4	4	**Q2**	3	3	3	3	3
	**Q3**	4	4	5	3	4	**Q3**	3	3	3	4	3
	**Q4**	5	5	5	4	5	**Q4**	3	5	5	3	5
	**Q5**	5	5	4	4	4	**Q5**	5	4	5	3	5
